# Phosphoserine phosphatase as an indicator for survival through potentially influencing the infiltration levels of immune cells in neuroblastoma

**DOI:** 10.3389/fcell.2022.873710

**Published:** 2022-08-26

**Authors:** Liang Zeng, Xiao-Yun Liu, Kai Chen, Liang-Jun Qin, Feng-Hua Wang, Lei Miao, Le Li, Hai-Yun Wang

**Affiliations:** ^1^ Department of Pathology, Guangzhou Women and Children’s Medical Center, Guangzhou Medical University, Guangdong Provincial Clinical Research Center for Child Health, National Children’s Medical Center for South Central Region, Guangzhou, China; ^2^ Department of Molecular Diagnostics, Sun Yat-Sen University Cancer Center, State Key Laboratory of Oncology in South China, Collaborative Innovation Center for Cancer Medicine, Guangzhou, China; ^3^ Departments of Thoracic Surgery, Guangzhou Women and Children’s Medical Center, Guangzhou Medical University, Guangdong Provincial Clinical Research Center for Child Health, National Children’s Medical Center for South Central Region, Guangzhou, China; ^4^ Department of Pediatric Surgery, Guangzhou Institute of Pediatrics, Guangdong Provincial Key Laboratory of Research in Structural Birth Defect Disease, Guangzhou Women and Children’s Medical Center, Guangzhou Medical University, Guangdong Provincial Clinical Research Center for Child Health, National Children’s Medical Center for South Central Region, Guangzhou, China

**Keywords:** neuroblastoma, PSPH, growth and metastasis, immune cell, prognosis

## Abstract

**Introduction:** Metabolic deregulation, a hallmark of cancer, fuels cancer cell growth and metastasis. Phosphoserine phosphatase (PSPH), an enzyme of the serine metabolism pathway, has been shown to affect patients’ prognosis in many cancers but its significance in neuroblastoma remains unknown. Here, we show that the functional role and potential mechanism of PSPH and it is correlated with survival of neuroblastoma patients.

**Patients and Methods:** The TARGET dataset (n = 151) and our hospital-based cases (n = 55) were used for assessing the expression level of PSPH associated with survival in neuroblastoma patients, respectively. Then, *in vitro* experiments were performed to define the role of PSPH in neuroblastoma. The ESTIMATE and TIMER algorithms were utilized to examine the correlation between PSPH expression level and abundance of immune cells. Further, Kaplan-Meier survival analysis was performed to evaluate the effect of both PSPH and immune cells on patients’ prognosis.

**Results:** High expression of PSPH was significantly associated with unfavorable overall survival (OS) and event-free survival (EFS) in both the TARGET dataset and our hospital-based cases, and was an independent predictor of OS (hazard ratio, 2.00; 95% confidence intervals, 1.21–3.30, *p* = 0.0067). *In vitro* experiments showed that high expression of PSPH significantly promoted cell growth and metastasis. Further, the ESTIMATE result suggested that high expression level of PSPH was negatively associated with low stromal and ESTIMATE score. Specifically, high PSPH expression was found to be negatively associated with CD8^+^ T cell, macrophages and neutrophils, which negatively affected survival of neuroblastoma patients (*p* < 0.0001, *p* = 0.0005, and *p* = 0.0004, respectively).

**Conclusion:** These findings suggested that PSPH expression could be a promising indicator for prognosis and immunotherapy in neuroblastoma patients by potentially influencing infiltration levels of immune cells.

## Introduction

Neuroblastoma (NB) originates from the sympathetic nervous system and accounts for more than 8% of malignancies in patients younger than 15 years and around 15% of all pediatric oncologic deaths (Maris et al., 2007). Despite intensive treatment involving surgical resection and chemoradiotherapy, it is often associated with poor clinical outcomes (He et al., 2020). Based on the patient’s age at diagnosis, tumor stage and MYCN amplification, NB cases are classified into a low-, intermediate-, or high-risk group following the International Neuroblastoma Risk Group (INRG) (Cohn et al., 2009). Generally, patients with NB diagnosed before the age of 18 months, at the early stage (stage 1, 2, or 4 s) and without MYCN amplification have better outcomes (Brodeur, 2003; Vo et al., 2014) while children with recurrent and/or metastatic NB have a dismal event-free survival (EFS, <25%). Due to the heterogeneity and divergent outcomes of NB, it is of great importance to identify better biomarkers for proper risk classification and prognostication to guide the clinical management of these patients and improve their outcomes.

Phosphoserine phosphatase (PSPH), an enzyme of the serine metabolism pathway, is located on the short arm of chromosome 7p11.2 and encodes PSPH which catalyzes the l-serine synthesis and lies downstream of PHGDH in the serine biosynthesis pathway (Mattaini et al., 2016). Serine biosynthesis has been reported to be increased in many tumors relative to matched normal tissues (Li et al., 2021). Studies have also indicated that increased PSPH expression was observed in many cancers including breast cancer, colorectal cancer, gastric cancer, hepatocellular carcinoma, melanoma, and non-small cell lung cancer (Liao et al., 2019; Rawat et al., 2021; Sun et al., 2016; D'Arcy et al., 2015; Allard et al., 2012; Sato et al., 2017; Sun et al., 2015). However, few studies have investigated the functional role of PSPH in NB.

The tumor microenvironment (TME) refers to the interactions between cancer cells and nonmalignant cells that affect the progression of tumors, composing of immune cells, blood vessels, extracellular matrix, fibroblasts, lymphocytes, and bone marrow-derived inflammatory cells (Arneth, 2019; Hinshaw and Shevde, 2019). It is commonly considered as immune ‘suppressive’ in most cancers mainly because the numbers of antigen-specific lymphocytes in tumors *per-se* are low (Van de Velde et al., 2021). Among these immune-related cells, T cells and macrophages are the most abundant. CD8^+^ T cells have been shown to have tumor-suppressive function while regulatory T cells have tumor-promoting functions. Macrophages also have various functions that are associated with cancer development and progression. However, the correlation between PSPH expression and immune cells infiltration has not been well-studied in NB.

In this investigation, we first collected the RNA sequencing data of NB samples from the Therapeutically Applicable Research to Generate Effective Treatments (TARGET) database to explore the association of PSPH expression level with NB patients’ survival, and the immune-infiltration abundance. Secondly, the ESTIMATE algorithm was used to assess the stromal and immune scores of NB samples, and thirdly, The ESTIMATE and TIMER algorithms were used to examine the relationship between PSPH expression and infiltration levels of specified immune cells.

## Patients and Methods

### Public raw data

The RNA sequencing data of 151 NB tissues were collected from the TARGET database (https://target-data.nci.nih.gov/Public/NBL/mRNA-seq/). The corresponding clinical data included age at diagnosis, the International Neuroblastoma Staging System (INSS), and MYCN amplified state.

### NB tissue

This study retrospectively recruited formalin-fixed paraffin-embedded (FFPE) tumour specimens from 55 NB patients treated at Guangzhou Women and Children’s Medical Center (GWCMC). The ethical statement was approved by the Institutional Ethics Committee of GWCMC (2021]078A01). This study was performed in accordance with the ethical standards of the World Medical Association Declaration of Helsinki.

### Immunohistochemistry

IHC staining was performed to detect PSPH expression in NB tissue samples. The IHC experiment was carried out according to our previous study (Wang et al., 2011). Briefly, the FFPE sections were rehydrated with a graded alcohol series. Endogenous peroxide activity was blocked with 3% hydrogen peroxide for 10 min at room temperature. The FFPE sections were boiled for antigen retrieval in tris (hydroxymethyl) aminomethane-ethylenediaminetetraacetic acid buffer (pH 8.0) in a pressure cooker for 2 min, 30 s and then incubated with indicated primary PSPH antibody in a 1:100 dilution (14513-1-AP, ProteinTech, Wuhan, China) overnight at 4°C in a humidified chamber. Reaction products were finally visualized by incubation with diaminobenzidine and the nucleus was counterstained with Meyer’s hematoxylin. Two pathologists (L.Z and K.C) who were blinded to the clinical features of the specimens independently scored the tissue slides based on PSPH expression using a semi-quantitative estimation. In details, the proportions of stained tumor cells were assigned as follows: 0, negative; 1, less than 30% of tumor cells stained; 2, more than 30% of tumor cells stained. Staining intensity score was designated as follows: 0, negative; 1, weak; 2, moderate and 3, strong. The multiple scores were divided as follows: 0 to two referred to a low score and three to six to a high score.

### Cell culture and cell transfection

The human NB cell lines SK-N-BE (MYCN amplified) and SK-N-SH (MYCN nonamplified) (Harenza et al., 2017) were obtained from the American Type Culture Collection (ATCC, Manassas, VA, United States). The cells were individually grown in RPMI-1640 medium and DMEM, supplemented with 10% fetal bovine serum (FBS) and 1% penicillin/streptomycin at 37 °C in 5% CO_2_. The cells were authenticated via short-tandem repeat profiling and tested free of *mycoplasma* contamination. pSLenti-EF1a-EGFP-P2A-Puro-CMV-MCS-3Flag and pSLenti-EF1a-EGFP-P2A-Puro-CMV-PSPH-3Flag expression constructs were purchased from OBiO Technology Inc (Shanghai, China). The constructs were transfected with 293T cells using Lipofectamine 3,000 (Invitrogen, Carlsbad, CA). Viral media were collected to infect SK-N-BE and SK-N-SH cells with polybrene (Santa Cruz, Biotechnology, Santa Cruz, CA) for 72 h. Cells with PSPH protein expression were evaluated by western blot.

### Western blot

Western blot was performed following standard procedures. Briefly, the treated cells were lysed in ice-cold RIPA buffer containing both protease and phosphatase inhibitors. Total protein concentration was then quantified using a BCA protein assay kit (Thermo Fisher Scientific, MA United States). The antibodies used were anti-Vinculin (#13901, Cell Signaling Technology, CST, Beverly, Massachusetts, United States), anti-Tubulin (#BS1482M, Bioworld, Nanjing, China), and anti-PSPH (14513-1-AP, Polyclonal, ProteinTech, Wuhan, China). All bands were visualized by enhanced chemiluminescence and quantified by ImageJ analysis software.

### Cell proliferation

Cell proliferation was measured by CCK8 assay following the manufacturer’s instructions (Dojindo.

Japan). SK-N-BE and SK-N-SH cells were plated onto 96-well plates for 24 h and treated as indicated. The cell viability index was obtained by calculating optical density (OD) values at 450 nm.

### Clone formation

SK-N-BE cells (1 × 10^3^/well) were plated into 6-wells supplemented with RMPI-1640 containing 10% FBS in 6-well plates. After incubation for 2 weeks, the cells were fixed with 100% methanol and stained with 0.5% crystal violet dye, after which the clones were visualized.

### Cell invasion and wound healing assay

For cell invasion assay, polycarbonate filter membranes with a diameter of 6.5 mm and pore size of 8 μm (Invitrogen) using membranes coated with Matrigel matrix were used (BDScience, Sparks, MD). Tumor cell suspensions (1 × 10^5^ cells/well) in serum-free culture medium were added to the upper chamber and incubated for 24 h at 37°C. The filters were fixed with methanol for 30 min and stained with 0.1% crystal violet for 20 min at room temperature. SK-N-BE and SK-N-SH cells were seeded into 6-well plates and incubated to reach 95% abundance for the wound healing assay and were analyzed using ImageJ software. The monolayer was scratched using a sterile 200 μl pipette tip and washed with a serum-free medium to remove detached cells. Cell motility in each group was assessed by visualizing the distance of open wound at indicated time intervals.

### Analysis of estimate score

Infiltrating stromal and immune cells constitute the major components of normal cells across tumour tissues, which not only disturb the tumour signal but also play an important role in tumorigenesis. ESTIAMTE algorithm (Estimation of STromal and Immune cells in MAlignant Tumour tissues) (Yoshihara et al., 2013) is a well-developed method that generates immune and stromal scores based on RNA-seq data. It is also combined the stromal and immune scores as the ‘ESTIMATE score’. In this study, the infiltration levels of stromal and immune cells in the TARGET dataset were determined via the “estimate” package of R software (https://R-Forge.R-project.org/projects/estimate/). A higher stromal or immune score represented a larger ratio of the corresponding component in the TME.

### Calculation of tumor-infiltrating immune cells

The Estimate model Tumor Immune Estimation Resource (TIMER) 2.0 was applied to analyze the infiltrating abundance of immune cells in all tumor samples (Li et al., 2016; Li et al., 2017; Li et al., 2020). Furthermore, the correlations of the PSPH expression level with the infiltrating immune cells were analyzed by Spearman correlation analysis, and the relationship between survival and infiltrating immune cells were also analyzed by survival package in R.

### Statistical analysis

All statistics analyses were performed using R (version 4.1.2), GraphPad Prism (version 8.0.1, San Diego, CA, United States), and Stata version 15.1 (Texas, United States). Summary statistics were provided for clinical and demographic characteristics. Correlations between clinical features and PSPH expression were analyzed with Pearson’s correlation analysis. All bar graphs show the mean ± SD and derived from three independent experiments with statistical significance using Student’s *t*-test (unpaired, two-tailed) or one-way ANOVA. The Survminer package (https://CRAN.R-project.org/package=survivalminer) was used to determine the cutoff values of PSPH expression, the ESTIMATE scores and the abundance of infiltrating immune cells. The Survival package (https://CRAN.R-project.org/package=survival) was used to plot the Kaplan-Meier curves for estimating overall survival (OS) and EFS among different groups with differences assessed by the log-rank test. The receiver operating characteristic curve (ROC) analysis was performed to calculate area under the curve (AUC) using the survival ROC package (https://CRAN.R-project.org/package=survivalROC). *p* value was set as 0.05 for indicating statistical significance.

## Results

### PSPH overexpression was independently associated with worse prognosis in NB patients

**FIGURE 1:**
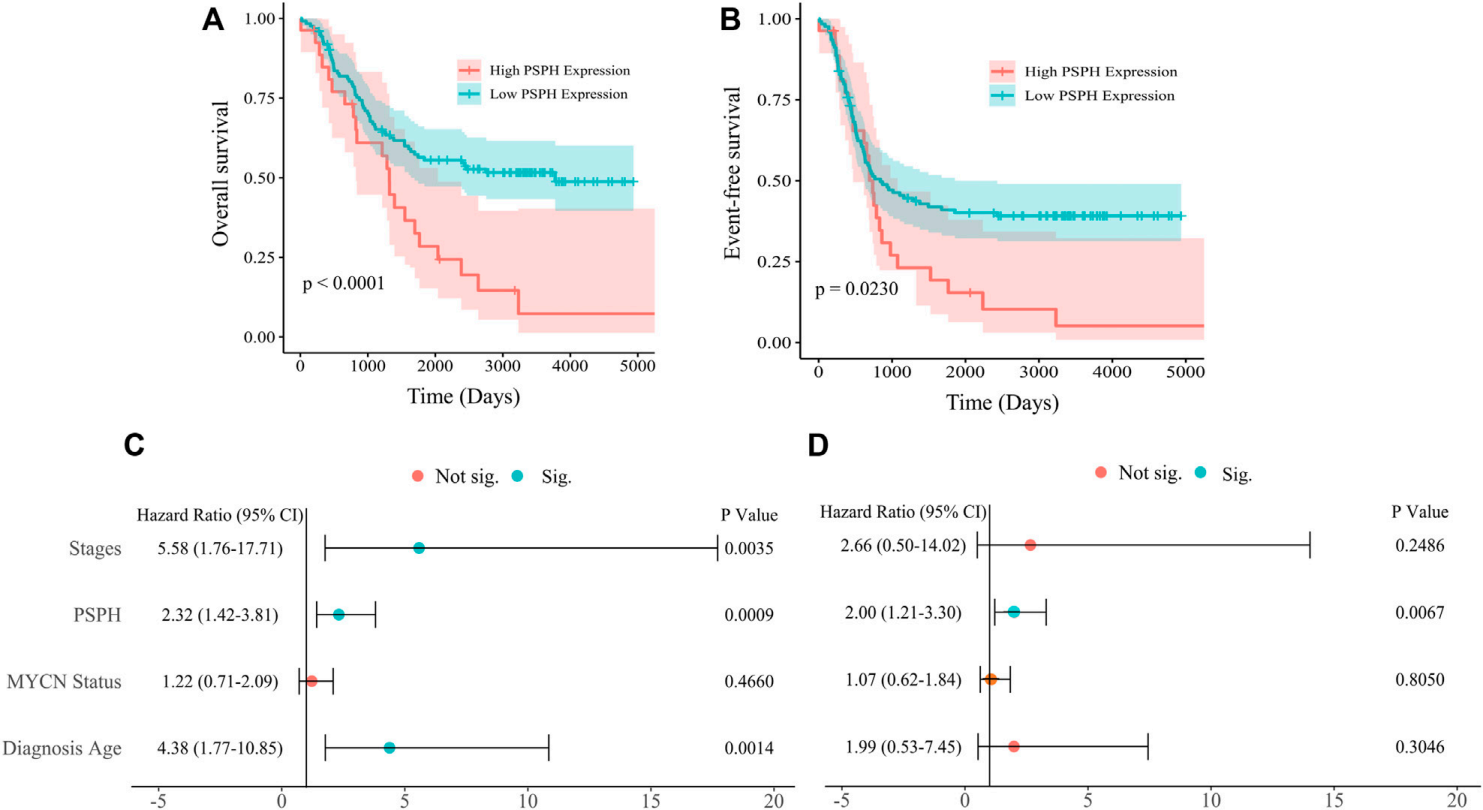
Survival analysis of 151 NB patients from the TARGET dataset. **(A,B)** Showing Kaplan-Meier survival curves of NB patients with different expression of PSPH (*p* < 0.0001 for OS; *p* = 0.0230 for EFS, respectively). **(C)** Univariate and **(D)** multivariate Cox analysis indicating that PSPH expression level was an independent risk factor for OS after adjusted with other risk factors such as INSS, MYCN amplified status and age at diagnosis. Green dot indicates significant.

**FIGURE 2:**
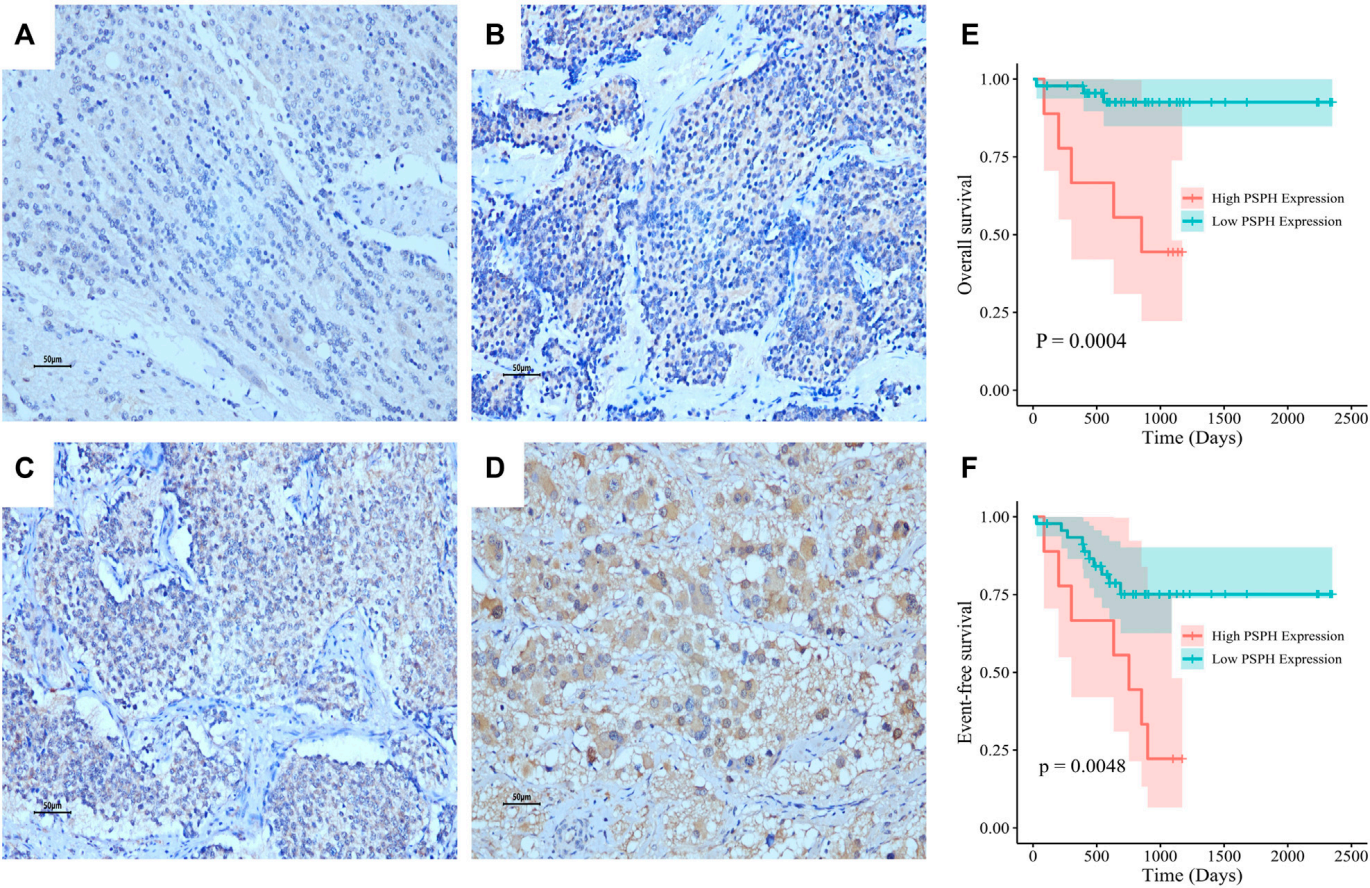
PSPH expression in NB tissue samples and the association with NB patients’ survival. Representative images of PSPH expression detected by IHC in NB patients: negative **(A)**, weak **(B)**, moderate **(C)** and strong staining **(D)**. The Kaplan-Meier survival of NB patients with the high and low expression of PSPH **(E)**, *p* = 0.0004 for OS; **(F)**, *p* = 0.0048 for EFS.

To assess the role of amino-acid metabolic gene PSPH in NB patients, the TARGET dataset was used to determine the association of PSPH expression with NB patients’ survival. Supplementary Table S1 shows the clinicopathological characteristics of NB patients in the TARGET dataset. Kaplan-Meier survival analysis indicated that high expression of PSPH was significantly associated with worse OS (hazard ratio [HR] 2.28, 95% confidence intervals [CI] 1.39–3.74, *p* < 0.0001, Figure 1A) and EFS (HR 1.73, 95% CI 1.04–2.87, *p* = 0.0230, Figure 1B). More importantly, high PSPH expression was identified as a risk factor in univariate Cox regression analysis (HR 2.32, 95% CI 1.42–3.81, *p* = 0.0009, Figure 1C), and subsequent multivariate Cox regression analysis indicated that high PSPH expression was an independent risk factor associated with poor prognosis in NB patients (HR 2.00, 95% CI 1.21–3.30, *p* = 0.0067, Figure 1D). ROC curve analysis also suggested that PSPH expression level had good performance for predicting the 1-, three- and 5-years OS rate of NB patients (Supplementary Figure S1, online only). Next, we examined the expression level of PSPH protein via IHC in NB FFPE samples. Table 1 shows the baseline characteristics and the representative staining images are displayed in Figures 2A–D. Similarly, Kaplan-Meier analysis showed that high PSPH expression was significantly associated with worse OS (*p* = 0.0004, Figure 2E) and EFS (*p* = 0.0048, Figure 2F). These results suggest that PSPH is an independent risk factor for prognosis in NB patients.

**TABLE 1:** Clinicopathologic characteristics of 55 neuroblastoma patients.

Characteristics	No. of patients (%)	PSPH expression		*p* value
		High	Low	
**Age (months)**				0.8610
<18	23 (41.8)	4	19	
≥18	32 (58.2)	5	27	
**Gender**				0.5820
Female	20 (36.4)	4	16	
Male	35 (63.6)	5	30	
**MYCN status**				0.2790
Amp	7 (14.6)	0	7	
Nonamp	41 (85.4)	6	35	
NA	7			
**Pathological grade**				0.9640
Differentiating	10 (24.4)	2	8	
Undifferentiated or Poorly Differentiated	31 (75.6)	6	25	
NA	14			
**INSS**				0.8380
Early	12 (22.2)	2	10	
Advanced	42 (77.8)	6	36	
NA	1			
**COG risk group**				0.9120
High	31 (63.3)	4	27	
Intermediate	7 (14.3)	1	6	
Low	11 (22.4)	2	9	
NA	6			
**3-years OS (95% CI)**	81.9% (66.1–90.8%)	44.4% (13.6–71.9%)	92.6% (78.6–97.6%)	
**3-years EFS (95% CI)**	61.3% (44.1–74.7%)	22.2% (3.4–51.4%)	75.1% (58.2–85.9%)	

### PSPH overexpression promoted cell proliferation and migration *in vitro*

**FIGURE 3:**
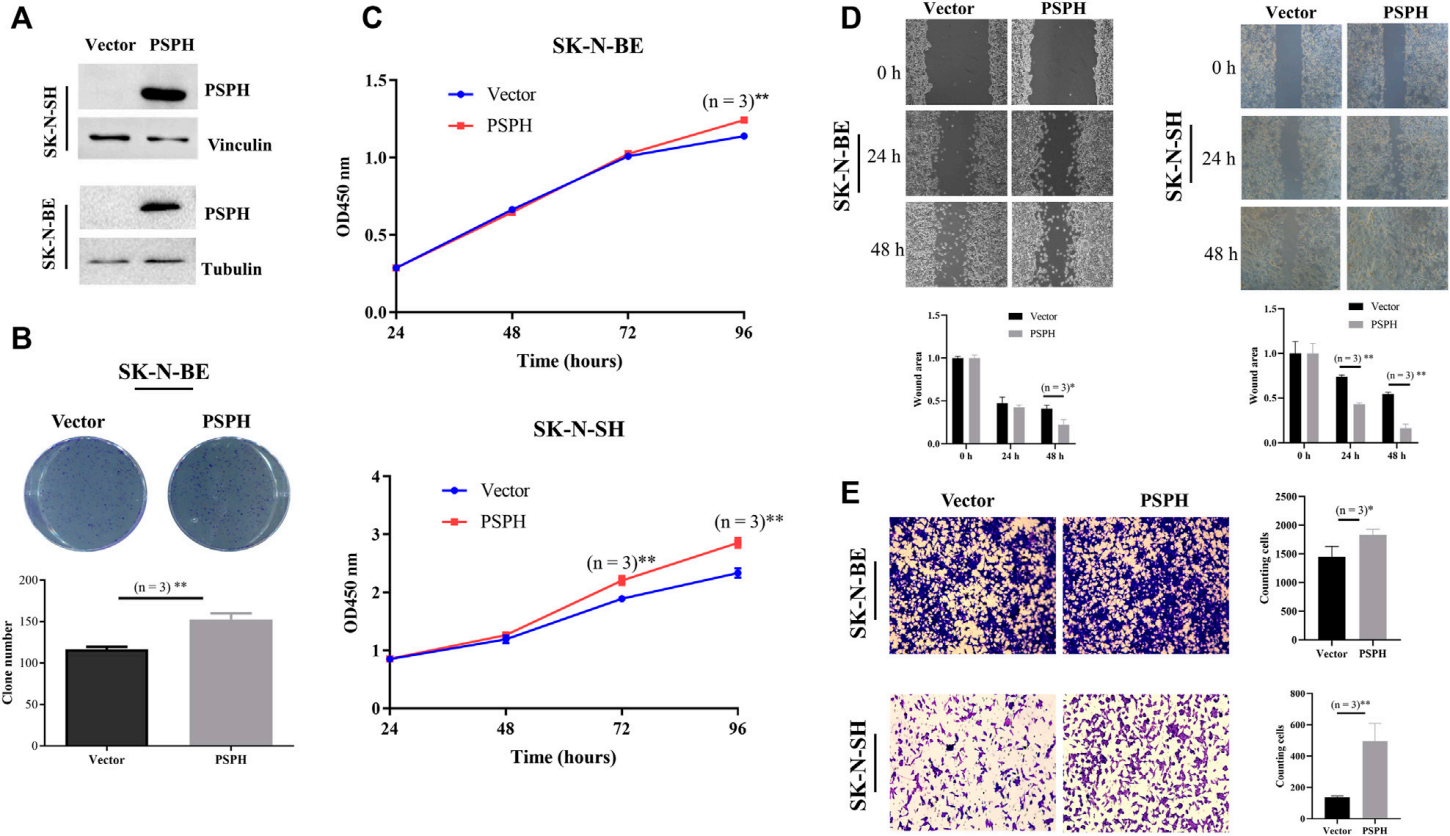
PSPH promoted cell proliferation and invasion of NB cells *in vitro*. **(A)** Western blot assay indicating the level of PSPH in two NB cell lines stably transfected with empty vector and PSPH (SK-N-SH and SK-N-BE); **(B)** Representative images of clone formation and statistical analysis indicating cell proliferation of human SK-N-BE cell treated as indicated; **(C)** CCK8 assay describing the changes of cell viability of SK-N-BE and SK-N-SH cells stably transfected with vector and PSPH treated as indicated; **(D)** Wound-healing assay of SK-N-BE and SK-N-SH cells exposing to vector and PSPH treated as indicated for 24 and 48 h independently; **(E)** Representative images (left panel) and quantification (right panel) of Matrigel invasion assay indicating the invasion ability of SK-N-BE and SK-N-SH cells stably transfected as indicated. The data in **(B–E)** are presented as mean ± SD of triplicate experiments, *p < 0.05, **p < 0.01.

To investigate the functional role of PSPH expression in NB patients, *in vitro* experiments were performed to assess the influence of PSPH expression on cell proliferation and migration in NB cell lines. The high expression level of PSPH in SK-N-BE and SK-N-SH cell lines were determined using western blot after transfection (Figure 3A). Subsequently, we examined the effects of PSPH on the phenotype of SK-N-BE and SK-N-SH cell lines. PSPH overexpression significantly promoted cell proliferation in SK-N-BE determined by clone formation assay (n = 3, *p* < 0.01, Figure 3B) and SK-N-BE and SK-N-SH cells determined by CCK8 assay (n = 3, *p* < 0.01, Figure 3C), respectively. Meanwhile, wound healing and cell migration assays revealed the increased cell mobility in SK-N-BE and SK-N-SH cells with PSPH overexpression (n = 3, *p < 0.05, **p < 0.01, Figures 3D,E). These findings show that PSPH affects NB cell malignant progression.

### Lower estimate and immune score in NB patients with high PSPH expression were correlated with worse prognosis

**FIGURE 4:**
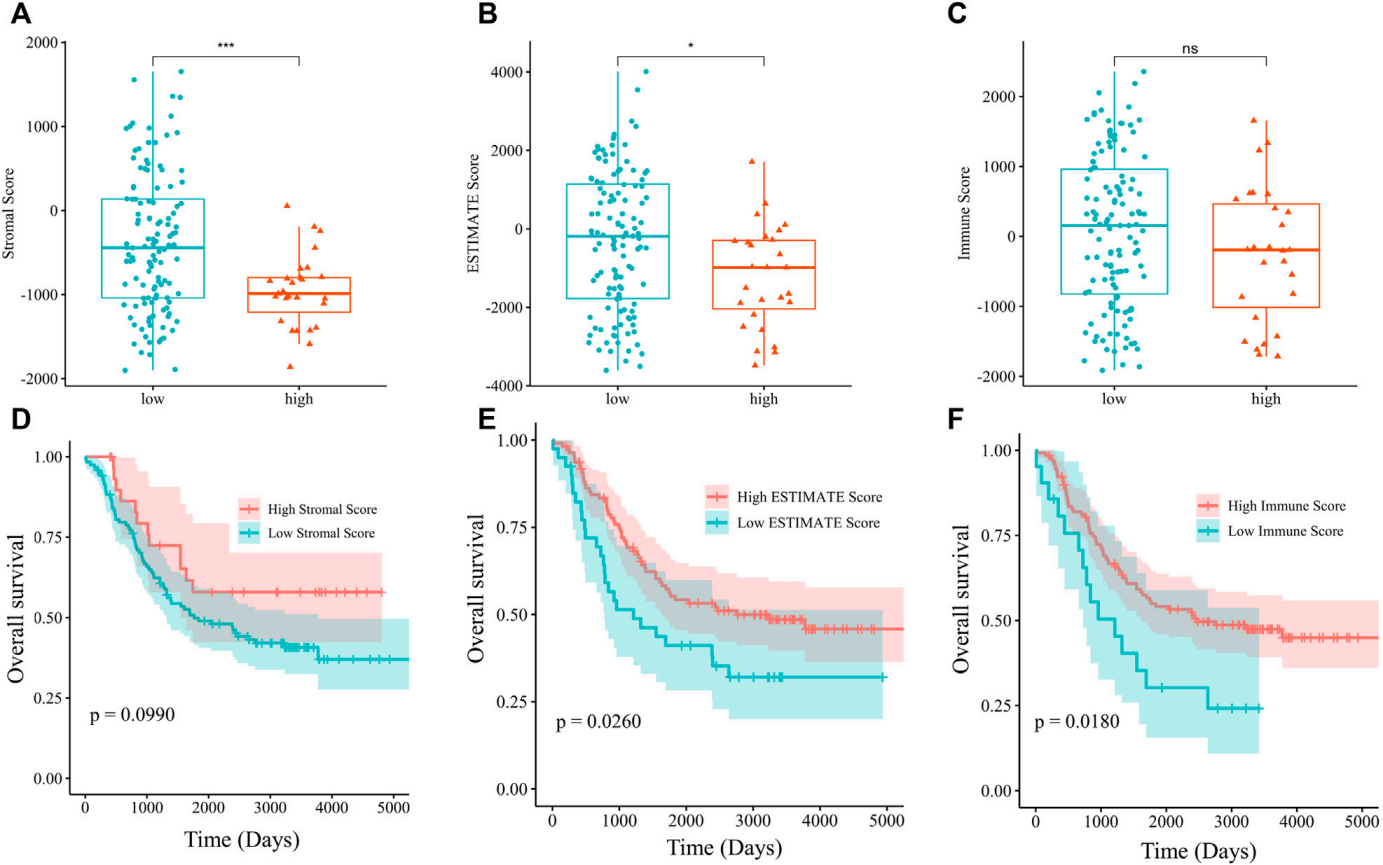
The analysis of relationship between the stromal **(A)**, ESTIMATE **(B)**, and immune score **(C)** and PSPH expression level. OS was evaluated by Kaplan-Meier method with log-rank test in NB patients grouped by the stromal **(D)**, ESTIMATE **(E)**, immune score **(F)**. *p < 0.05, ***p < 0.001.

We hypothesized that PSPH regulating serine-dependent pathways were affected in the TME, and this phenomenon could affect T cell phenotypic states which are linked to the inability of the host to control tumor progression. Since we have already shown that PSPH expression significantly contributed to the patients’ poor survival and tumour cell progression, we then explored the potential mechanisms of PSPH in NB. The ESTIMATE algorithm was used to assess the infiltration levels of immune cell in the TARGET dataset. Accordingly, the significant difference was observed in the stromal score (*p* < 0.001, Figure 4A) and ESTIMATE score (*p* < 0.05, Figure 4B) between NB patients with high- and low-PSPH expression. To establish the linkage between ESTIMATE score and the NB patients’ survival, Kaplan–Meier plots were generated. Survival analysis indicated that the low ESTIMATE and immune scores had unpreferable OS performance, with *p* value of 0.0260, and 0.0180, respectively (Figures 4E,F). These data demonstrate PSPH resulting in poor survival might be associated with the infiltration levels of immune cells in the TME.

### CD8^+^ T cells, macrophages and neutrophils infiltration levels were negatively correlated with PSPH expression and poor prognosis

**FIGURE 5:**
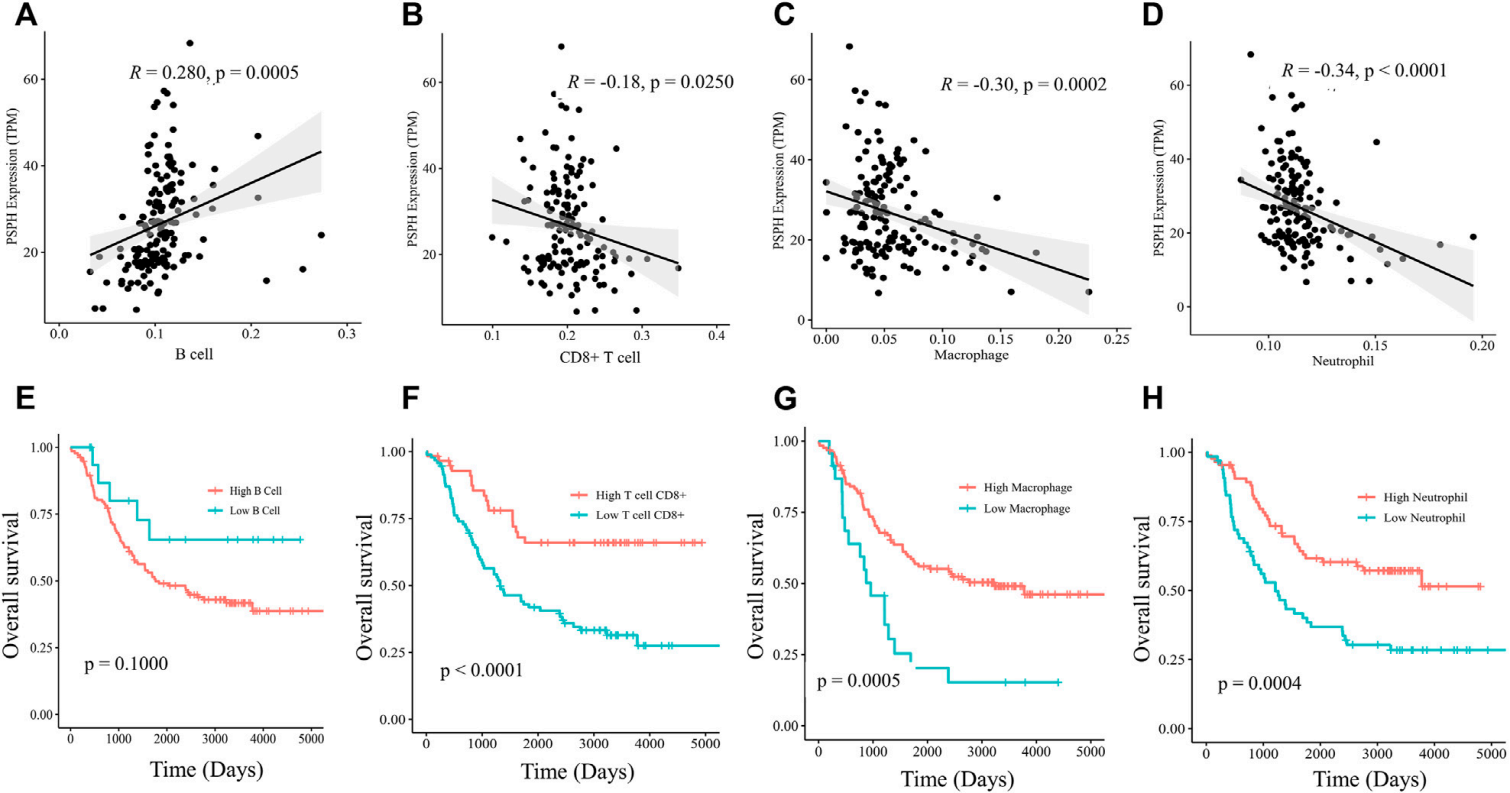
Scatter plots of B cell **(A)**, CD8^+^ T cell **(B)**, macrophages **(C)**, neutrophils **(D)** and PSPH expression in NB patients. Kaplan-Meier survival plots with log-rank test of different infiltration levels of B cell **(E)**, CD8^+^ T cell **(F)**, macrophages **(G)** and neutrophils **(H)**, and their association with the OS of NB patients.

To delineate the immune cell types involving the mechanisms of PSPH, the infiltration abundance of specified immune cells in the 151 samples were estimated by the Estimate model in TIMER 2.0. Specifically, PSPH expression was found to be positively correlated with B cells (R = 0.280, *p* = 0.0005, Figure 5A), but negatively correlated with CD8^+^ T cells, macrophages, and neutrophils (R = -0.18, *p* = 0.0250, Figure 5B; R = -0.30, *p* = 0.0002, Figure 5C; and R = -0.34, *p* < 0.0001, Figure 5D, respectively). Moreover, survival analysis performed on these immune cells showed that lower infiltration levels of CD8^+^T cell, macrophages, and neutrophils remarkably affected the prognosis (*p* < 0.0001, Figure 5F; *p* = 0.0005, Figure 5G; and *p* = 0.0004, Figure 5H, respectively). No significant correlation between the infiltration levels of CD4^+^ T cell and myeloid dendritic cells with PSPH expression was observed (Supplementary Figure S2, online only). These results show that PSPH expression level is negatively associated with CD8^+^ T cell, macrophages, and neutrophils in the TME, partially accounting for PSPH contributing to poor prognosis.

## Discussion

In this study, we found that high expression of PSPH was associated with poor prognosis and was an independent prognostic factor for OS in NB patients. The ESTIMATE algorithm indicated the potential mechanisms with high expression of PSPH accompanied by a lower stromal score, along with poor prognosis. We further found that PSPH was negatively related to CD8^+^ T cell, macrophages, and neutrophils and survival analysis indicated that lower infiltration levels of these cells were significantly associated with poor prognosis of NB patients. To the best of our knowledge, this is the first study identifying amino-acid metabolic gene PSPH associated with a worse prognosis in neuroblastoma, possibly along with infiltration levels of certain immune cells in the TME.

Multiple complex stromal and immune components in the TME are capable of suppressing and promoting tumour progression. In the current study, we identified TME features in the TARGET database via ESTIMATE and TIMER. The former calculated the score of immune and stromal components, and the latter identified the close correlation between PSPH expression and a particular subtype of immune cells. The TIMER showed that PSPH expression level was negatively related with CD8^+^ T cell, macrophages, and neutrophils. Additionally, we found that their lower infiltration levels were closely associated with unfavorable survival in patients with NB. This is in line with other previous studies. Several studies have demonstrated that high levels of CD8^+^ T cell was associated with favorable survival in cancers such as hepatocellular carcinoma, breast cancer, and ovarian cancer (Consortium et al., 2017; Savas et al., 2018; Guo et al., 2020). In NB patients, Wei et al. reported that higher abundance of CD8^+^ T cell was positively associated with long-term survival (Wei et al., 2018). Zarour et al. explained this phenomenon could be probably due to tumors escaping from the host immunity upon exhaustion of CD8^+^ T cell (Zarour, 2016).

Macrophages are major components of the TME and have a pivotal role in shaping the TME and tumor immunity. On the one hand, tumor-associated macrophages are closely associated with the tumor malignant progression. It has been reported that the infiltration of macrophages might result in CTLA4-mediated immune suppression and lead to poor prognosis in glioblastoma patients (Guan et al., 2021). Asgharzade et al. reported the infiltration of macrophages in metastatic NB (Asgharzadeh et al., 2012). On the other hand, high macrophage densities have been shown to be correlated with increased survival in non-small cell lung cancer (Kim et al., 2008). In our study, it was found that PSPH expression was adversely correlated with high macrophages densities in NB patients, who had favorable survival.

It is well known that active neutrophils participate in innate and adaptive immune responses via diverse mechanisms. What’s more, the role and importance of neutrophils in cancers is being increasingly appreciated. The dual impact of neutrophils through secretion of reactive oxygen species, autophagy and modulation of other immune cells supported or inhibited cancer development (Shaul and Fridlender, 2021). A recent study has identified that longer progression-free survival was observed in non-small cell lung cancer patients with higher percentages of neutrophils (Zamora Atenza et al., 2022). Additionally, affluent serine management can suppress inflammatory responses by increasing glutathione synthesis in certain animal experiments (Knudsen and Rorsman, 2019). We have shown that high neutrophil infiltration was associated with favorable survival in NB patients with low PSPH expression. Integrative findings suggest that metabolic gene PSPH predicted survival by regulating the infiltration densities of immune cells.

Accordingly, these above findings suggest that PSPH expression is closely linked to NB immune cell infiltration and thus, might affect the TME and be a prognostic biomarker in NB. These findings underscore the correlation of PSPH expression with different types of immune cell infiltration especially CD8^+^ T cell, macrophages, and neutrophils. Therefore, the increased PSPH expression might dysregulate the infiltration of immune cells involving immunity suppression, leading to a poor prognosis in NB patients.

Nevertheless, there were several limitations in this study. We did not perform *in vivo* and *in vitro* experiments to deeply explore mechanisms of PSPH regulating infiltration levels of immune cells. Future studies are therefore needed to validate our findings. However, it still remains interesting to incorporate information on the presence of PSPH expression in the context of immune cells, which will likely lead to better understanding of the functional role of poor survival in NB.

In summary, these results show that the important of PSPH in neuroblastoma growth and metastasis and prognosis prediction. PSPH expression levels impact the TME of NB, especially CD8^+^ T cell, macrophages, and neutrophils. Altogether, PSPH might be a potential target in reversing immune escape and provide new insights into understanding the function of PSPH expression in NB prognosis and tumor immunology.

## Data Availability

The original contributions presented in the study are included in the article/Supplementary Material, further inquiries can be directed to the corresponding author.

## Abbreviations

AUC, area under the curve; CI, confidence interval; EFS, event-free survival; FFPE, formalin-fixed paraffin-embedded; FBS, fetal bovine serum; GWCMC, guangzhou women and children’s medical center; HR, hazard ratio; INRG, international neuroblastoma risk group; INSS, international neuroblastoma staging system; IHC, immunohistochemistry; NB, neuroblastoma; OS, overall survival; PSPH, phosphoserine phosphatase; ROC, receiver operating characteristic; TARGET, therapeutically applicable research to generate effective treatments; TME, tumor microenvironment; TIMER, tumor immune estimation resource
