# Supplementary material for: Phosphoserine phosphatase as an indicator for survival through potentially influencing the infiltration levels of immune cells in neuroblastoma
Source: Front Cell Dev Biol. 2022 Aug 26;10:873710. doi: 10.3389/fcell.2022.873710 (PMC9459050; doi:10.3389/fcell.2022.873710)
Supplement: Supplementary file 1 [file DataSheet2.PDF]

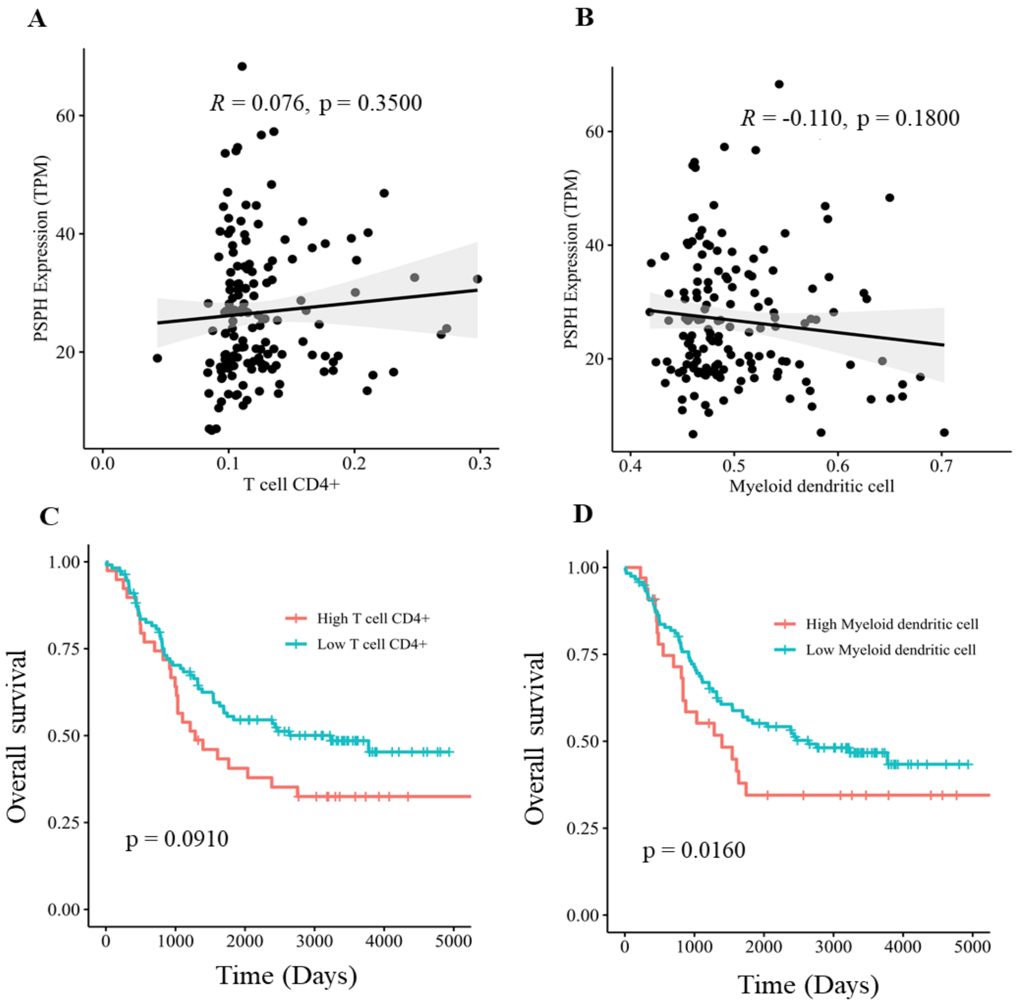

Supplementary Figure S2. The scatter plots of T cell CD4^{+} (**A**), myeloid dendritic cell (**B**), and PSPH expression in NB patients. And Kaplan-Meier plots with log-rank test analysis of different of T cell CD4^{+} (**C**), myeloid dendritic cell (**D**), and evaluating OS in NB patients.

Abbreviations: PSPH, phosphoserine phosphatase; NB, neuroblastoma; OS, overall survival.
