# Supplementary material for: Phosphoserine phosphatase as an indicator for survival through potentially influencing the infiltration levels of immune cells in neuroblastoma
Source: Front Cell Dev Biol. 2022 Aug 26;10:873710. doi: 10.3389/fcell.2022.873710 (PMC9459050; doi:10.3389/fcell.2022.873710)
Supplement: Supplementary file 2 [file DataSheet3.PDF]

Supplementary Table S1. Clinicopathologic characteristics of 151 NB patients from the TARGET dataset

Characteristics	No. of patients (%)	PSPH expression		*p* value
		Low (n=124)	High (n=27)	
**Age (months)**				0.273
< 18	28 (18.5)	25 (20.2)	3 (11.1)	
≥ 18	123 (81.5)	99 (79.8)	24 (88.9)	
**Gender**				0.639
Female	62 (41.1)	52 (41.9)	10 (37.0)	
Male	89 (58.9)	72 (58.1)	17 (63.0)	
**MYCN status**				0.132
Amp	32 (21.2)	30 (24.2)	2 (7.4)	
Nonamp	118 (78.2)	93 (75.0)	25 (92.6)	
NA	1 (0.6)	1 (0.8)		
**Pathological grade**				0.932
Differentiating	9 (6.0)	7 (5.7)	2 (7.4)	
Undifferentiated or Poorly Differentiated	118 (78.2)	97 (78.2)	21 (77.8)	
NA	24 (15.8)	20 (16.1)	4 (14.8)	
**INSS**				0.574
Early	22 (14.6)	19 (15.3)	3 (11.1)	
Advanced	129 (85.4)	105 (84.7)	24 (88.9)	
**COG risk group**				0.542
High	124 (82.1)	100 (80.6)	24 (88.9)	
Intermediate	13 (8.6)	12 (9.7)	1 (3.7)	
Low	14 (9.3)	12 (9.7)	2 (7.4)	
**3-year OS (95% CI)**	65.8% (57.5%-72.9%)	66.8% (57.7%-74.5%)	60.9% (39.5%-76.8%)	
**3-year EFS (95% CI)**	48.0% (39.3%-56.2%)	52.4% (42.8%-61.3%)	26.9% (11.0%-45.8%)	

Abbreviations: PSPH, phosphoserine phosphatase; INSS, the International Neuroblastoma Staging System; COG, Children's Oncology Group; OS, overall survival; EFS, event-free survival; CI, confidence interval; NA, not available.
