# Supplementary material for: Phosphoserine phosphatase as an indicator for survival through potentially influencing the infiltration levels of immune cells in neuroblastoma
Source: Front Cell Dev Biol. 2022 Aug 26;10:873710. doi: 10.3389/fcell.2022.873710 (PMC9459050; doi:10.3389/fcell.2022.873710)
Supplement: Supplementary file 3 [file DataSheet1.PDF]

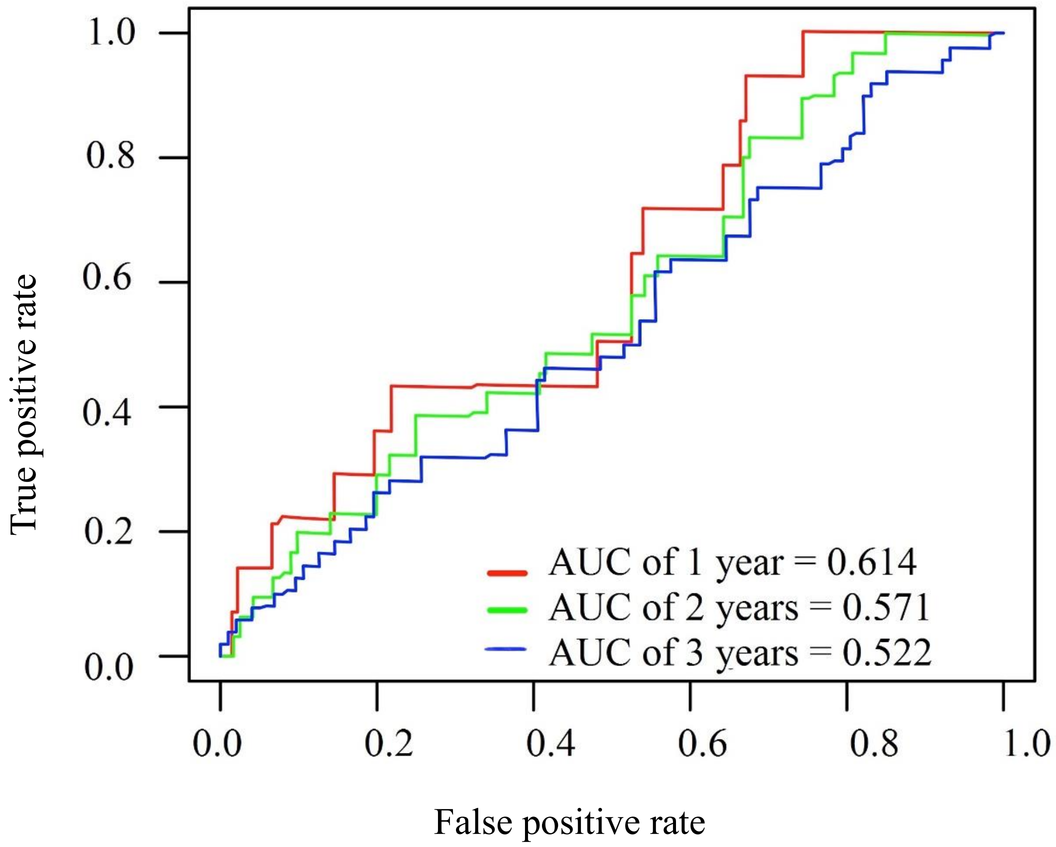

Supplementary Figure S1. Receiver operating characteristic (ROC) curves for PSPH expression as predictors of death in TARGET NB patients within 1 year, 2 years and 3 years. Abbreviations: ROC, receiver operating characteristic; PSPH, phosphoserine phosphatase; NB, neuroblastoma.
